# Comparative Analysis of Symmetry Parameters in the E2 Inner Core of the Pyruvate Dehydrogenase Complex

**DOI:** 10.3390/ijms252413731

**Published:** 2024-12-23

**Authors:** Han-ul Kim, Myeong Seon Jeong, Mi Young An, Yoon Ho Park, Sun Hee Park, Sang J. Chung, Yoon-sun Yi, Sangmi Jun, Young Kwan Kim, Hyun Suk Jung

**Affiliations:** 1 Department of Biochemistry, College of Natural Sciences, Kangwon National University, Chuncheon 24341, Republic of Korea; 2 Kangwon Center for Systems Imaging, Chuncheon 24341, Republic of Korea; 3 Center for Bio-Imaging Translational Research, Korea Basic Science Institute, Cheongju 28119, Republic of Korea; 4 School of Pharmacy, Sungkyunkwan University, Suwon 16419, Republic of Korea; 5 AbTis Co., Ltd., Suwon 16648, Republic of Korea

**Keywords:** cryo-electron microscopy, single-particle analysis, structural dynamics, macromolecule, pyruvate dehydrogenase complex

## Abstract

Recent advances in cryo-electron microscopy (cryo-EM) have facilitated the high-resolution structural determination of macromolecular complexes in their native states, providing valuable insights into their dynamic behaviors. However, insufficient understanding or experience with the cryo-EM image processing parameters can result in the loss of biological meaning. In this paper, we investigate the dihydrolipoyl acetyltransferase (E2) inner core complex of the pyruvate dehydrogenase complex (PDC) and reconstruct the 3D maps using five different symmetry parameters. The results demonstrate that the reconstructions yield structurally identical 3D models even at a near-atomic structure. This finding underscores a crucial message for researchers engaging in single-particle analysis (SPA) with relatively user-friendly and convenient image processing software. This approach helps reduce the risk of missing critical biological details, such as the dynamic properties of macromolecules.

## 1. Introduction

Cryo-electron microscopy (cryo-EM) has made significant contributions to the field of structural biology, particularly since nearly a decade ago during the so-called “resolution revolution” [1,2]. This transformative period was marked by the advent of direct electron detectors (DEDs), high-end transmission electron microscopy (TEM), and, simultaneously, the development of software packages for image processing [3,4]. These advancements collectively propelled cryo-EM into becoming a groundbreaking method in structural biology, allowing for unprecedented insights into molecular structures and biological phenomena [5,6]. Although traditional methods in structural biology have made significant contributions over the decades, they offer limited abilities to conduct comprehensive structural determinations of relatively high-molecular-weight (MW) proteins, protein complexes, particularly large macromolecules, and viruses [7]. Consequently, methods that complement the shortcomings of established techniques, such as X-ray crystallography and nuclear magnetic resonance (NMR), have received considerable attention [8].

From the perspective of structural biology, which includes the study of structural dynamics, analyzing the structures of proteins in their native state is crucial [9,10]. Moreover, a comprehensive structural analysis of the target in its native complex state can provide valuable information about the inherent protein–protein interactions (PPIs) of the protein or protein complex [11]. Understanding PPIs is crucial to grasp both their functionality and stability [12]. Additionally, an understanding of these factors can provide important insights not only into the protein structures but also their own structural specification [13]. In this regard, the most widely used method for the high-resolution structural analysis of proteins using cryo-EM is single-particle analysis (SPA) [14]. SPA is a powerful approach for obtaining near-atomic-resolution structural information about proteins or macromolecules [15,16]. Owing to the numerous image processing software packages developed over the past few decades, SPA has become relatively straightforward to use to deliver rapid and excellent results [17,18]. However, given the enhanced accessibility and user-friendliness of image processing procedures for SPA, users should strive to exclude subjective opinions and maximize the utilization of structural information to achieve the actual reconstruction of protein structures [19,20].

With the aim of proposing an approach to improve the processing results, we focused on the difference among the various symmetry parameters in SPA to analyze the dihydrolipoyl acetyltransferase (E2) inner core complex of the pyruvate dehydrogenase complex (PDC) and verified the results based on these symmetry parameters. We study the influence of different symmetry parameters on the 3D reconstruction of the E2 complex by using five parameters: asymmetry (C1), cyclic5 (C5), dihedral2 (D2), tetrahedral (T), and icosahedral (I). These findings convey an important message about the details users might overlook in the 3D reconstruction of a dynamic protein structure using SPA with cryo-EM. Ultimately, the accurate application of symmetry parameters is essential not only for obtaining better maps but also for enhancing our understanding of structural dynamics, thereby facilitating a deeper analysis of the biological relevance of protein assemblies.

## 2. Results

### 2.1. Structural Features of the E2 Complex of B. stearothermophilus

**Figure 1:**
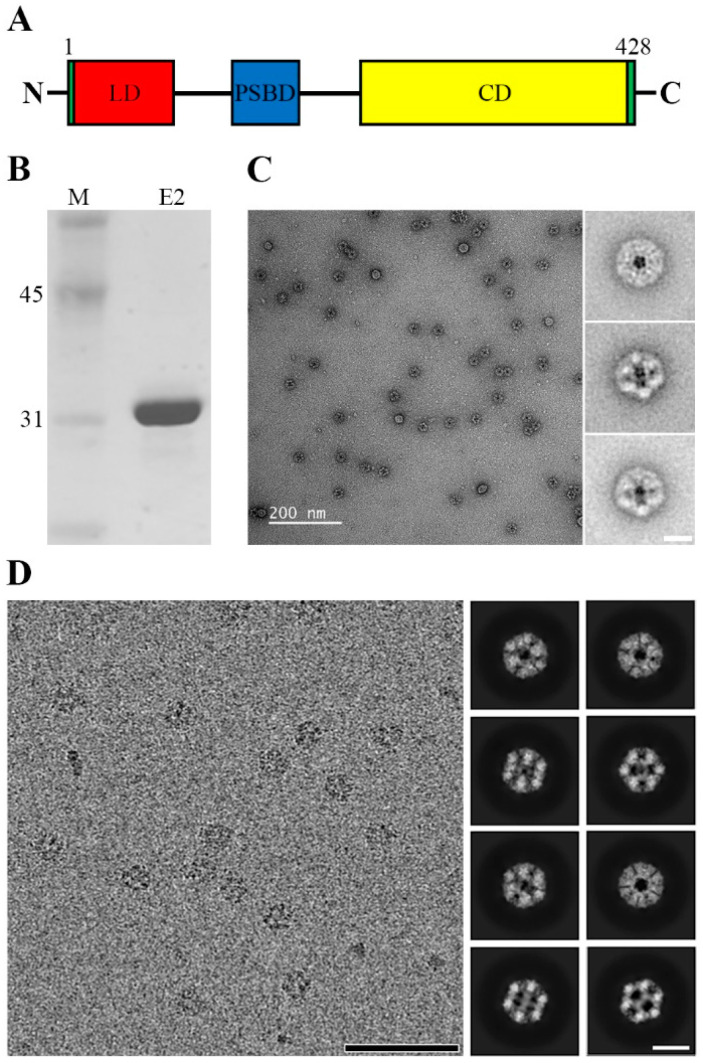
Electron micrographs of the purified E2 complex. (**A**) Composition of the monomeric domain of the E2 inner core complex of the PDC. N, N-terminus; C, C-terminus; LD, lipoyl binding domain; PSBD, peripheral subunit binding domain; CD, core domain. (**B**) Proportion of the purified supernatant E2 complex in SDS-PAGE. M, Marker (31 and 45 kDa). (**C**) Fields of negatively stained (**left**) and selected 2D averages (**right**) of the purified E2. (**D**) Cryo-EM micrograph (**left**) and selected final 2D averages (**right**) from classified particles of the frozen hydrated E2 complex. Scale bars in (**C**), 200 nm (**left**) and 25 nm (**right**); (**D**), 200 nm (**left**) and 20 nm (**right**).

The E2 protein of *B. stearothermophilus* is a protein with an MW of approximately 43 kDa. It is a highly conserved protein comprising the lipoyl-binding domain (LD, 2-77) located in the N-terminal region, the peripheral subunit-binding domain (PSBD, 130-167) situated in the middle region, and the catalytic acetyltransferase core domain (CD, 214-425) positioned in the C-terminal region (Figure 1A) [21]. To enhance the yield of protein expression and complex formation through *E. coli*, we constructed a region containing only the CD by including amino acid 185 to 425, but excluding the LD and PSBD [22,23]. As shown in Figure 1B, we confirmed the monomeric form at approximately 32 kDa on SDS-PAGE. The purified truncated E2 was observed to appropriately form complexes in the negatively stained microscopic field; we observed a structure similar to that of several previously published E2 complexes, as depicted in Figure 1C. Additionally, through unstained frozen hydrated micrographs for 3D reconstruction of the E2 complex using cryo-EM, we confirmed a size of approximately 25 nm. As a result, by utilizing 2D classification, we observed the E2 complex in high resolution from various angles, as illustrated in Figure 1D.

### 2.2. Reconstruction of the E2 Complex, Determined Using Various Applied Symmetry Parameters

**Figure 2:**
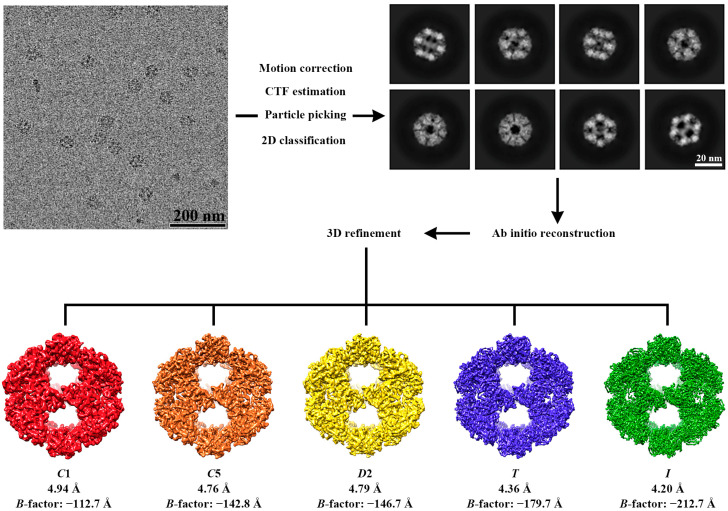
Workflow of cryo-EM data processing. Summarized workflow of data pre-processing (motion correction, CTF estimation, and particle picking), 2D classification, and 3D refinement for each reconstruction.

**Figure 3:**
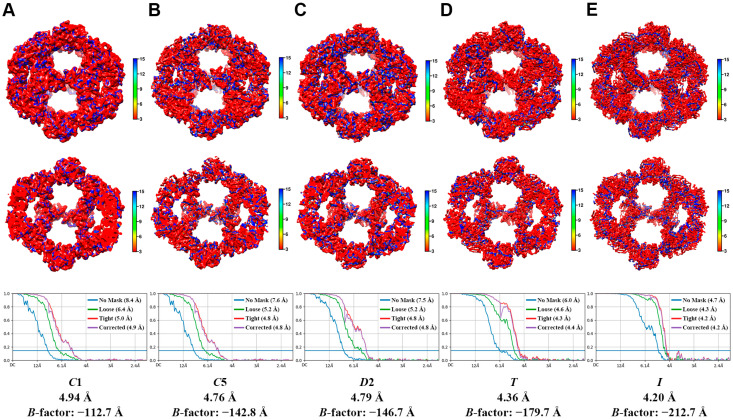
Local and global resolution estimates for cryo-EM maps. (**A**), C1; (**B**), C5; (**C**), D2; (**D**), T; (**E**), I, respectively. Local resolution estimations are illustrated by variation in surface color (top and middle) and were calculated using CryoRes. Global resolution (bottom) was estimated at FSC = 0.143 from half maps and were estimated using CryoSPARC.

**Figure 4:**
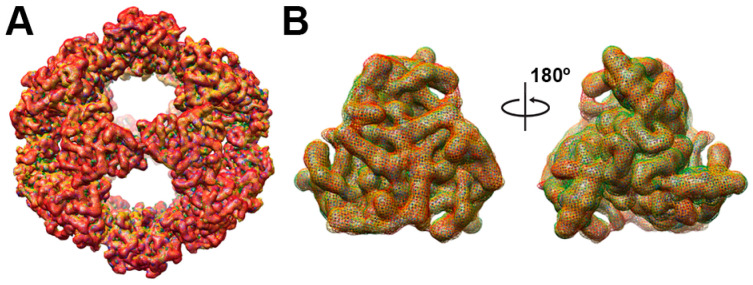
Illustrated comparison of reconstructed cryo-EM maps derived from different symmetric restraints. (**A**) Overall deviations among the five superposed 3D densities, represented by the mesh envelope, corresponding to each symmetric restraint: C1 (red), C5 (orange), D2 (yellow), T (blue), and I (green). (**B**) Enlarged view of the segmented region of each trimeric unit from each model that constitutes the respective map shown (**A**).

The 3D reconstruction of E2 was performed to investigate the impact of the symmetry parameters on the image processing results. For this purpose, we used 2D classification to select a final particle set consisting of 69,946 particles. Using the model generated by the same ab initio reconstruction as the initial model, we carried out the process to determine the values of five different parameters: C1, C5, D2, T, and I. The final version of the E2 cryo-EM model achieved the highest resolution through reconstruction using the I parameter, and each reconstruction yielded the following resolution values: C1 = 4.93 Å, C5 = 4.76 Å, D2 = 4.79 Å, T = 4.36 Å, and I = 4.20 Å (Figure 2 and Figure 3). The units constituting the entire complex were consistently reconstructed identically in all five models. Many previous studies on the structural analysis of various E2 complexes predominantly utilized the symmetric restraint I for reconstruction [22,24,25]. However, our results demonstrated that not only this symmetric restraint, which is commonly considered, but also parameters such as C1, C5, D2, and T are similar to the previous results of the E2 complex. The comparative results in Figure 4A, superposed based on C1 as the center, are displayed to illustrate these findings. Due to slight differences in the resolution, it is not possible to discern the differences in perfect detail. However, it can be acknowledged to a satisfactory extent that the five maps were reconstructed into the same 3D shapes. Moreover, for a more detailed comparison, the E2 trimeric complex, which is composed of three E2 monomers, was segregated to ascertain that, in combination, their 3D structures in fact constituted that of the entire trimeric complex (Figure 4B).

### 2.3. Comparison of the Pseudo-Atomic Models of the E2 Complex with C1 Cryo-EM Map

**Figure 5:**
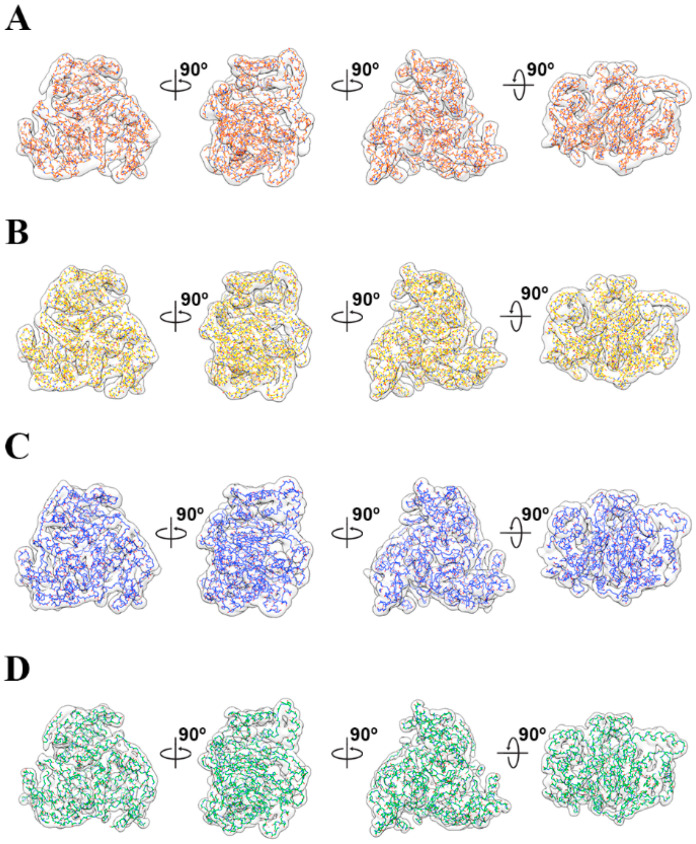
Pseudo-atomic models of each symmetric reconstruction. (**A**–**D**) Atomic models calculated from each trimeric EM map for detailed comparison. The models for C5, D2, T, and I are colored orange, yellow, blue, and green, respectively. Each atomic model is shown by fitting the envelope of the corresponding EM envelope. The model is rotated by 90° around the x- and y-axes in (**A**–**D**), respectively.

**Figure 6:**
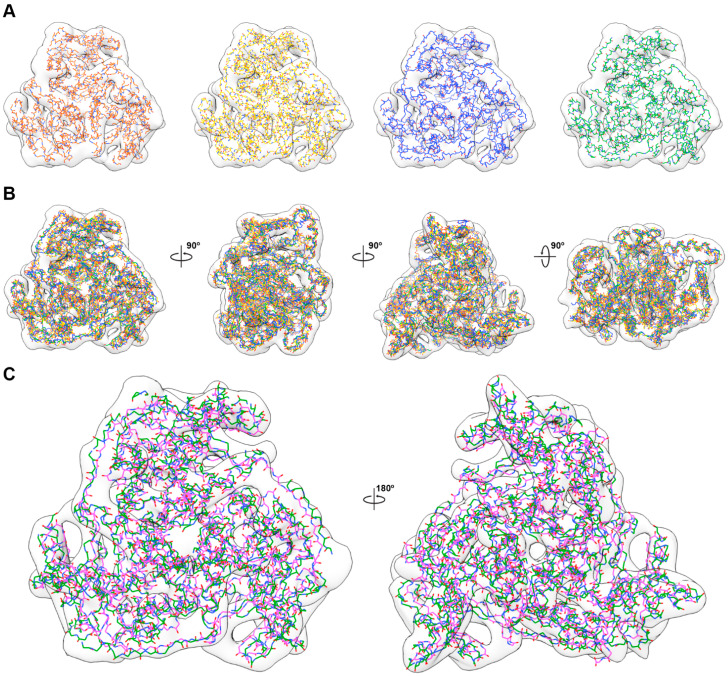
Comparison of each atomic model with the C1 cryo-EM map. (**A**) Shows the superposition of each pseudo-atomic model excluding C1 on to the C1 trimeric complex form of cryo-EM map. (**B**) Combined pseudo-fitted results of all calculated models onto the C1 cryo-EM envelope for comprehensive comparison. The model is rotated by 90° around the x- and y-axes. (**C**) Comparison of the pseudo-fitting of the atomic model generated by I symmetric restrains and the trimeric EM envelope of C1 with the PDB: 1B5S. The respective models are colored as follows: C5, orange; D2, yellow; T, blue; I, green; 1B5S, magenta.

Next, we conducted a more detailed comparison by performing atomic model building using each model, except for the C1 map. Atomic models of C5, D2, T, and I were built using ModelAngelo [26], which employs sequence-based machine learning to reconstruct each model (Figure 5A–D). The structure of each atomic model corresponds well with the cryo-EM map of C1, which serves as crucial evidence that the individual reconstructions, including that of C1, are similar structures (Figure 6A). Furthermore, to determine whether structural differences exist among the different models (excluding C1), we superposed the different models based on the C1 cryo-EM map (Figure 6B). This result confirmed that the respective atomic models did not structurally differ from one another. Subsequently, to compare with previously published results, we examined the structural differences by fitting our pseudo-atomic model with the highest resolution, I, with the model from PDB-1B5S, onto the C1 map (Figure 6C). This demonstrates that there are no significant structural differences.

## 3. Discussion

Deciphering the molecular symmetry in cryo-EM is indeed valuable, especially in SPA, a widely adopted approach for the structural analysis of biological samples. Thus, reliable structural analysis through SPA can provide a deeper understanding of the physiological phenomena such as PPIs and the physiological meaning of these interactions. It offers informative insight into protein assembly resulting from the structural dynamics of proteins. Moreover, the dynamics involved in protein assembly and interaction are critical for their functionality and can influence how proteins respond to environmental changes.

Thus, we compared the results of the E2 complex by applying parameters, such as I, C5, D2, T, and C1. However, due to the 3D models corresponding to a resolution of 4.20 to 4.93 Å, detailed structural analysis of the E2 complex at the atomic level was not possible. Next, although our results consistently reveal structurally identical 3D maps, it has been observed that the resolution gradually decreases compared to I. If the E2 complex adheres perfectly to icosahedral symmetry, employing C1, C5, D2, and T as symmetry parameters in the context of cryo-EM is essentially equivalent to a reduction in the number of particle images of the E2 complex because C1, C5, D2, and T are in fact sub-groups of I symmetry [27,28]. Thus, when considering I encompasses 60 elements, C1, comprising only 1 element, results in a 1/60 reduction. D2, consisting of 4 elements, leads to a 1/15 reduction. C5, with 5 elements, corresponds to a 1/12 reduction. Nevertheless, we confirmed through pseudo-atomic modeling that, despite the use of different parameters, all exhibited the same 3D structure in terms of the structural aspect.

Therefore, a lack of understanding or inexperience with the parameters used for processing could result in vital aspects being overlooked and, ultimately, flawed results. This approach helps to mitigate the risk of overlooking crucial aspects from a biological perspective. Information shown in this study can convey an important message to researchers who study high-resolution protein structures and the structural dynamics of a protein complex.

## 4. Materials and Methods

### 4.1. Protein Expression and Purification

The deoxyribonucleic acid (DNA) encoding E2 protein Bacillus stearothermophilus (*B. stearothermophilus*) was amplified by polymerase chain reaction (PCR) using suitable primers and the corresponding cDNA (UniProt: P11961) as templates, and the PCR products were digested and inserted into the *pET 30a* (+) vector (Merck Millipore, Darmstadt, Germany), which had been digested with the corresponding restriction enzymes. The forward and reverse primers used were P1 and P2 for SpyTag-PDH, P1 (BamHI site underlined): 5′-CGC GGA TCC GCT CAT ATT GTT ATG-3′, P2 (XhoI site underlined): 5′-CGG CTC GAG TTA TTA ATA ATA ATT-3′. The prepared DNA was transformed into *Escherichia coli* (*E. coli*) One ShotTM BL 21 StarTM (DE3) (Thermo fisher Scientific, Waltham, MA, USA). The expression of recombinant E2 was induced by adding 0.1 mmol/L isopropyl-β-D-thiogalactoside (IPTG) at 18 °C for 16 h [29]. Cells were harvested by centrifugation (2808× *g* at 4 °C for 15 min), washed with a buffer (50 mM Tris pH 7.5, 500 mM NaCl, 5% glycerol, and 1 mM phenylmethylsulfonyl fluoride (PMSF)), and lysed by ultra-sonication. After centrifugation (30,438× *g* at 4 °C for 25 min), the supernatant was purified by heat precipitation for 20 min at 70 °C. After heating, samples were centrifuged (30,438× *g* at 4 °C for 25 min) [22,30].

### 4.2. Conventional Transmission Electron Microscopy

The condition of the purified proteins was confirmed with heavy-metal staining. A 5 μL sample of the E2 complex (100 nM) was applied to freshly glow-discharged (Harric Plasma, Ithaca, NY, USA) carbon-coated copper grids. The grids were then negatively stained with 1% uranyl acetate [31] and examined under a JEM-2100F electron microscope (JEOL, Tokyo, Japan) operated at 200 kV. Images were captured with a 4K Gatan Oneview CMOS camera at a nominal magnification of 40,000 (0.28 nm/pixel). Then, the 2D class averages were generated from 1887 particles using the EMAN2 package to enhance the signal-to-noise ratio (SNR) of the negatively stained data for visual inspection [32].

### 4.3. Cryo-EM Sample Preparation

A sample (4 μL of 100 nM) was applied to a holey carbon grid (Quantifoil R2/R2 100 mesh, Au NH2 finder, EMS, Santa Fe Springs, CA, USA), which had been negatively glow-discharged at 15 mA for 1 min using easiGlow (PELCO, Fresno, CA, USA). The grid was blotted for 5 s with force −1 Ted Pella 595 filter paper (Ted pella, Redding, CA, USA) using Vitrobot Mark IV (Thermo Fisher Scientific, USA) at 4 °C with 100% humidity and then quickly plunged frozen in liquid ethane cooled by liquid nitrogen. Instrumentation was used at the Kangwon Center for Systems Imaging (Chuncheon, Republic of Korea) and Korea Basic Science Institute (Daejeon, Republic of Korea).

### 4.4. Data Collection and Image Processing

Automated data collection was performed using EPU on a Talos Artica G2 transmission electron microscope (Thermo Fisher Scientific, USA) operating at 200 kV, equipped with a K3 detector. Each movie was recorded with a total dose of 40 e-/Å-2 per movie and a defocus range of −1.8 to −2.6 μm. The movies were collected in electron counting mode with a pixel size of 1.1 Å. A total of 5250 movies were acquired, and before conducting the 3D reconstruction, motion correction was estimated, and the contrast transfer function (CTF) was adjusted. After estimation of the 5250 micrographs, the particles were picked from micrographs without templates (Blob picking). A total of 90,756 particles were extracted. Of these, 69,946 particles were then selected based on visual inspection from the best-matched classes resulting from 2D classification. Next, five initial reference models were created with heterogeneous ab initio reconstruction. Subsequently, using the same initial volume, homogeneous 3D refinement was conducted with the C1, C5, D2, T, and I symmetry parameters. The final model of each symmetry parameter was refined with a soft-edge mask and sharpened with B-factors of −112.7, −142.8, −146.7, −179.7, and −212.7 Å, respectively. All image processing procedures were carried out using CryoSPARC [33]. The resolution of the final cryo-EM map was estimated by gold-standard Fourier shell correlation (FSC) (0.143 criterion) between the two halves of the dataset within CryoSPARC. The local resolution was calculated with CryoRes, a deep learning-based direct estimation [34]. The processing procedure is described in Figure 2, and the data collection and refinement statistics for the cryo-EM are summarized in Table 1. The final maps were deposited with the Electron Microscopy Data Bank (EMDB) (EMD-38333, C1; EMD-38334, C5; EMD-38335, D2; EMD-38340, T; EMD-38336, I;).

### 4.5. Model Building

A pseudo-atomic model of each model was built using the automated model-building tool, modelAngelo [26]. Based on the map densities and sequence, the sharpened EM maps were segmented into trimeric complexes using UCSF Chimera [35], and each atomic model was automatically calculated using machine learning based on the sequence of PDB-1B5S. The modeling statistics were validated using PHENIX [36] (Table 1), and all structural analyses were conducted using UCSF Chimera.

## Figures and Tables

**Table 1:** Data collection and refinement statistics.

Data Collection					
Dataset	C1 EMD-38333	C5 EMD-38334	D2 EMD-38335	TEMD-38340	IEMD-38336
Particles	69,946	69,946	69,946	69,946	69,946
Pixel size (Å)	1.1	1.1	1.1	1.1	1.1
Defocus range (μm)	−1.5 to −2.6	−1.5 to −2.6	−1.5 to −2.6	−1.5 to −2.6	−1.5 to −2.6
Total electron dose (e-/Å^2^)	40	40	40	40	40
Refinement					
Resolution (Å)	4.93	4.76	4.79	4.36	4.20
Map-sharpening B-factor (Å)	−112.7	−142.8	−146.7	−179.7	−212.7
Average B-factor (Å)	−132.7	−162.8	−166.7	−199.7	−232.7
RMSD					
Bond length (Å)		0.155	0.156	0.118	0.109
Bond angle (°)		18.492	19.857	12.514	11.822
Ramachandran plot					
Favored (%)		43.31	41.57	67.23	70.75
Allowed (%)		19.48	20.90	16.29	13.19
Disallowed (%)		37.21	37.53	16.48	16.06
MolProbity score		5.12	5.23	4.65	4.55

## Data Availability

The datasets used and/or analyzed during the current study are available from the corresponding author upon reasonable request.
